# The switch of DNA states filtering the extrinsic noise in the system of frequency modulation

**DOI:** 10.1038/s41598-021-95365-0

**Published:** 2021-08-11

**Authors:** Shih-Chiang Lo, Chao-Xuan You, Bo-Ren Chen, Ching-Chu Hsieh, Cheng-En Li, Che-Chi Shu

**Affiliations:** Department of Chemical Engineering and Biotechnology, National Taipei University of Technology, No.1, Sec. 3, Chung-Hsiao E. Road, Taipei City, 10608 Taiwan

**Keywords:** Transcription, Gene regulatory networks

## Abstract

There is a special node, which the large noise of the upstream element may not always lead to a broad distribution of downstream elements. This node is DNA, with upstream element TF and downstream elements mRNA and proteins. By applying the stochastic simulation algorithm (SSA) on gene circuits inspired by the *fim* operon in *Escherichia coli*, we found that cells exchanged the distribution of the upstream transcription factor (TF) for the transitional frequency of DNA. Then cells do an inverse transform, which exchanges the transitional frequency of DNA for the distribution of downstream products. Due to this special feature, DNA in the system of frequency modulation is able to reset the noise. By probability generating function, we know the ranges of parameter values that grant such an interesting phenomenon.

## Introduction

The intracellular noise is ubiquitous and it presents phenotypic variation to genetically identical cells^1^. The expression variability arises not only from the stochastic nature of the very process, but also from other processes which cause fluctuations in the number of cellular molecules such as regulatory proteins. Researchers in recent decades investigated how the inherited noise affects biological reaction networks. Pedraza and Oudenaarden^2^ monitored three genes in Escherichia coli by fluorescent proteins and observed the noise propagation from upstream elements to downstream proteins. Hooshangi et al.^3^ discovered an ultrasensitive cascade in noise propagation. Wu et al.^4^ examined the role of reaction rate on filtering the upstream noise. Others studied the connection between pathway and noise propagation^5–7^. Our goal is to explore the function of DNA in noise propagation.

The cell-to-cell variation plays a critical role in determining cellular behaviors, such as the detection of signal^8,9^, the switch of intracellular states^10–14^, and so on. Normally, increased phenotypic variation helps cells adapt to the fluctuating environments^15–17^, and decreased phenotypic variation benefits cells in constant environments^18^. Given the importance of phenotypic variation, controlling the noise in gene expression is critical to cells^19^. Ozbudak et al.^20^ proposed the most well-known method of tuning noise. By varying the binding sequence of the ribosome, the increment of translational efficiency increases the noise intensity at the protein level. Others reported that the TATA box is associated with high noise in eukaryotic gene expression^21,22^. Still others, recently through a mathematical model, found that protein–ligand interaction^23^ and the reaction of dimerization^24^ are noise-buffering motifs. Also, it is possible to suppress noise by introducing control elements; both the negative feedback control^25,26^ and the incoherent feedforward loop (FFL)^27,28^ attenuate noise.

Different types of mechanisms by which transcription factors (TFs) modulate the dynamics of transcription have distinctive ways of noise propagation. In a system of amplitude modulation (AM), TFs enhance the rate of transcription and control the amplitude of transcription burst. We will briefly go through the noise propagation in the system of AM in this study, but we are interested in the system of frequency modulation (FM) where the TF shortens the lifetime of DNA state and thus modulate frequency^29^. In FM, many research articles discussed the systems without concerning the noise of the TF^30^. They include the studies examining how the switching of DNA affects the downstream elements^19,31,32^. Without a doubt, the upstream TF may disturb the distribution of downstream elements. A detailed discussion of how the TF affects the DNA was overlooked for decades. We dealt with the FM system in which DNA has only two states, active and inactive. If we count the active state as one and the inactive zero, the mean value over the population automatically reveals the variance as well as all other higher moments. Due to this unique feature, it is impossible to store the information of the TF noise by the distribution of the DNA states. It must have other ways to propagate information. In the present study, we aim to unveil the mystery of DNA, as a special node in gene regulation, being super powerful in blocking the noise of upstream elements.

## Results

### The reaction network

**Figure 1:**
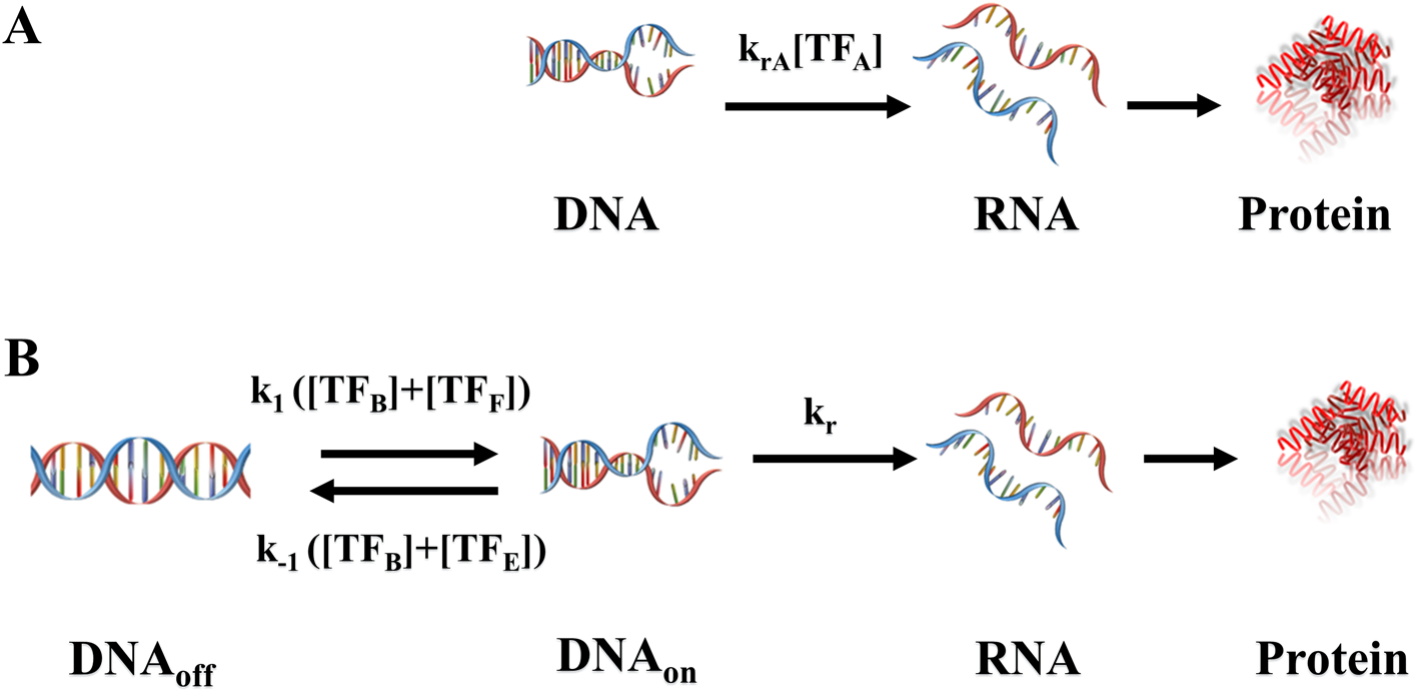
(**A**) In the system of amplitude modulation (AM), the transcription factor controls the transcription rate. (**B**) In the system of frequency modulation (FM), the transcription factors decide the flipping rate of DNA configuration.

Figure 1A,B are the gene expression through amplitude and frequency modulation of TF, respectively. Both systems include TFs acting on the DNA. In the AM system (Fig. 1A), the increment of TF_A_ gradually increases the expression. On the other side, in the FM system (Fig. 1B), inspired by FimE and FimB in *E coli*^19^, we accounted for TF_B_ which switches DNA configuration in both directions at equal rates and TF_E_ increases k_off_, the rate constant switching DNA from ON to OFF. To have a better understanding, we also included hypothetic TF_F_ which increases k_on_, the rate constant switching DNA from OFF to ON. In the present study, we first examined the AM system and then the FM system.

Note that the processes from DNA to the downstream protein in the FM system were well studied in the literature^19,31^. Hung et al.^19^ experimentally demonstrated that the switching of DNA remarkably affects the noise at the protein level. However, few researchers discussed the noise propagation from TFs to downstream protein. In the present study, we aim to unveil the extraordinary role of DNA in noise propagation and demonstrate how it reset the noise.

### The noise at the protein level inherited the noise of TF_A_

**Figure 2:**
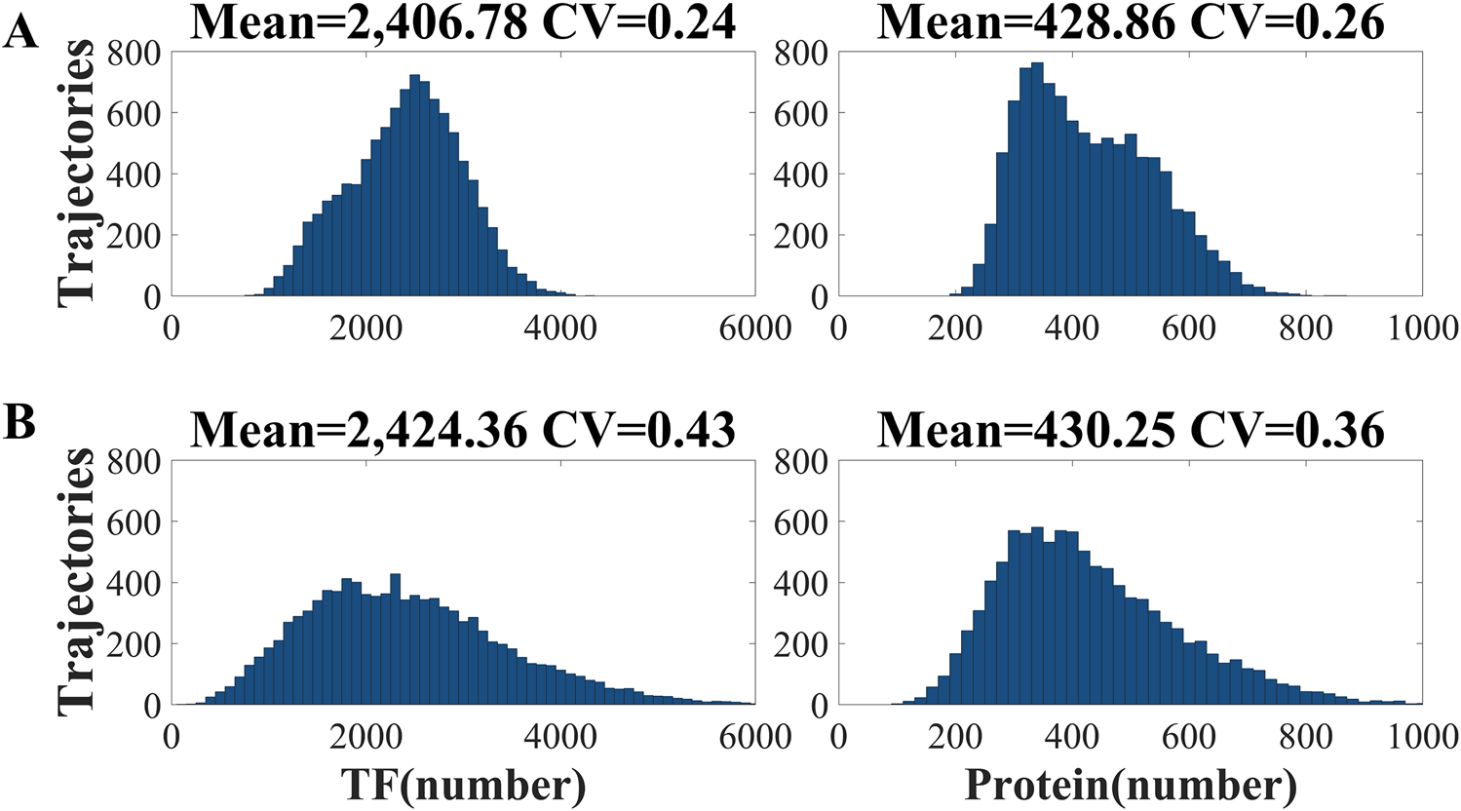
The noise propagation in the AM system. The x-axis is the expression level of TF_A_ (left panels) or downstream protein (right panels). The y-axis is the number of trajectories (cells) having the TF_A_ or protein level indicated by the x-axis. (**A**) Sharp distribution of TF_A_ leads to low noise intensity at the protein level. (**B**) High CV of TF_A_ results in broad distribution at the protein level.

We first examined how the noise of TF_A_ affects the downstream protein in the AM system (Fig. 1A). Figure 2A demonstrates the distribution of TF_A_ (left panel) and the downstream protein (right panel). Figure 2A,B show the system with low and high noise of TF_A_, respectively. In comparison to Fig. 2A,B shows that the broad distribution of TF_A_ led to the broad distribution at the protein level. The coefficient of variation (CV) in Fig. 2 also supports that the downstream protein inherited the noise of TF_A_.

### The noise propagation was blocked in the FM system

**Figure 3:**
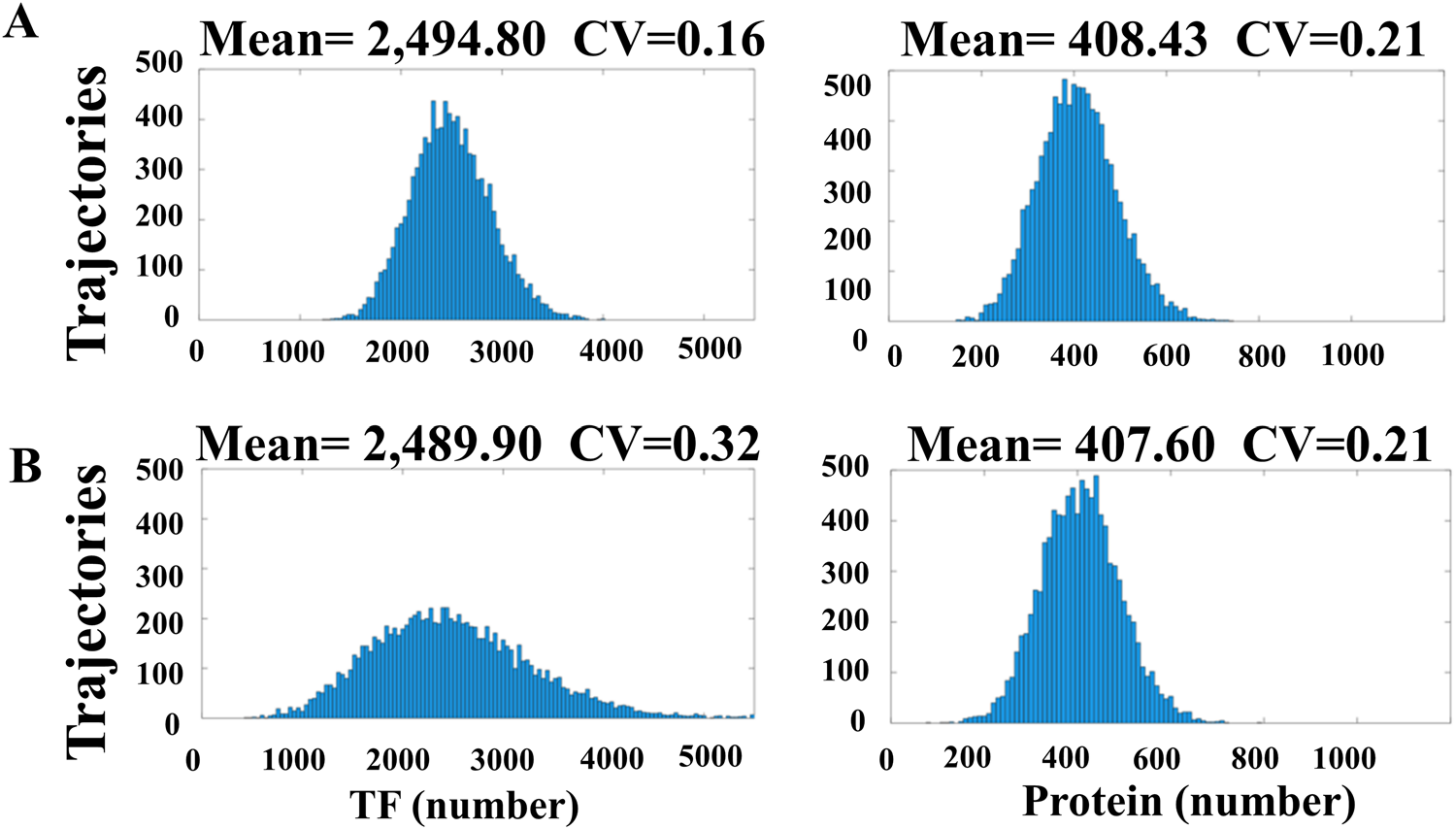
The noise propagation in the FM system. The x-axis is the expression level of TF_B_ (left panels) or downstream protein (right panels). The y-axis is the number of trajectories (cells) having the TF_B_ or protein level indicated by the x-axis. In the FM system, the noise of TF has little effect on the distribution of the downstream protein. Both (**A**) narrow distribution and (**B**) broad distribution of TF lead to almost the same distribution at the protein level.

We then examined how the noise of TF_B_ affects the downstream protein in the FM system (Fig. 1B). Figure 3A demonstrates the distribution of TF_B_ (left panel) and the protein (right panel). The distribution of TF_B_ (left panel) in Fig. 3A is much sharper than that in Fig. 3B. If the downstream protein inherits the upstream noise, the protein distribution (right panel) in Fig. 3A should be much narrower than that in Fig. 3B. However, the CV of protein in Fig. 3A,B are almost the same. The result suggests that the upstream noise is blocked out.

### The role of DNA in noise propagation

**Figure 4:**
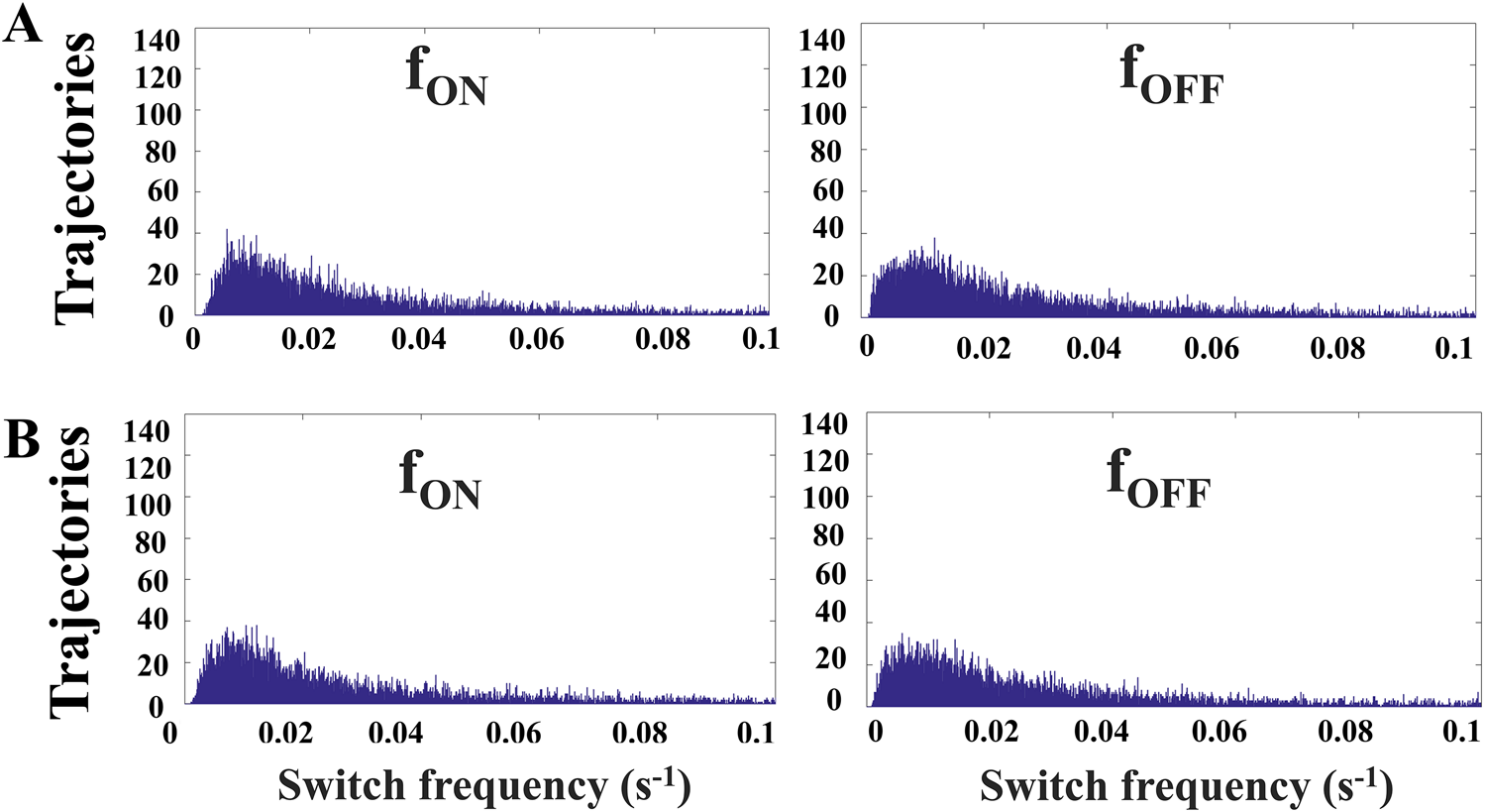
The f_ON_ represents the reciprocal of the time required from the OFF state to the ON state and f_OFF_ is from the ON to the OFF. We discuss the switching frequency of DNA for two cases in Fig. 3A,B. There are two noise intensities of TF, (**A**) low and (**B**) high. Interestingly, both of them have nearly the same distribution of f_ON_ and f_OFF_.

The bizarre noise propagation in Fig. 3 results from the unique feature of DNA. There are only two states of DNA, the active and inactive conformations, and the distribution in population merely describes the ratio of these two states. If we count the active state of DNA as one and the inactive state as zero, the values of all moments are automatically determined provided that the mean value is known. Clearly, the information regarding the distribution of upstream TF_B_ is NOT recorded by the states of DNA in the population. Instead, DNA intriguingly uses the switching frequency to pass the information. In Fig. 4A, the f_ON_ (left panel) represents the reciprocal of the time required for DNA switching from the inactive state to the active state and the f_OFF_ (right panel) is that from the active state to the inactive state. Figure 4A,B illustrated the switching frequency for the cases of Fig. 3A,B, respectively. The distribution of switching frequency in Fig. 4A is almost the same as that in Fig. 4B, so is the distribution of the downstream protein in Fig. 3A almost the same as that in Fig. 3B. Interestingly, the information of upstream TF_B_ is transformed into the switching frequency of DNA, f_ON_ and f_OFF_. Then the switching frequency is reversely transformed into the distribution of the downstream element.

**Figure 5:**
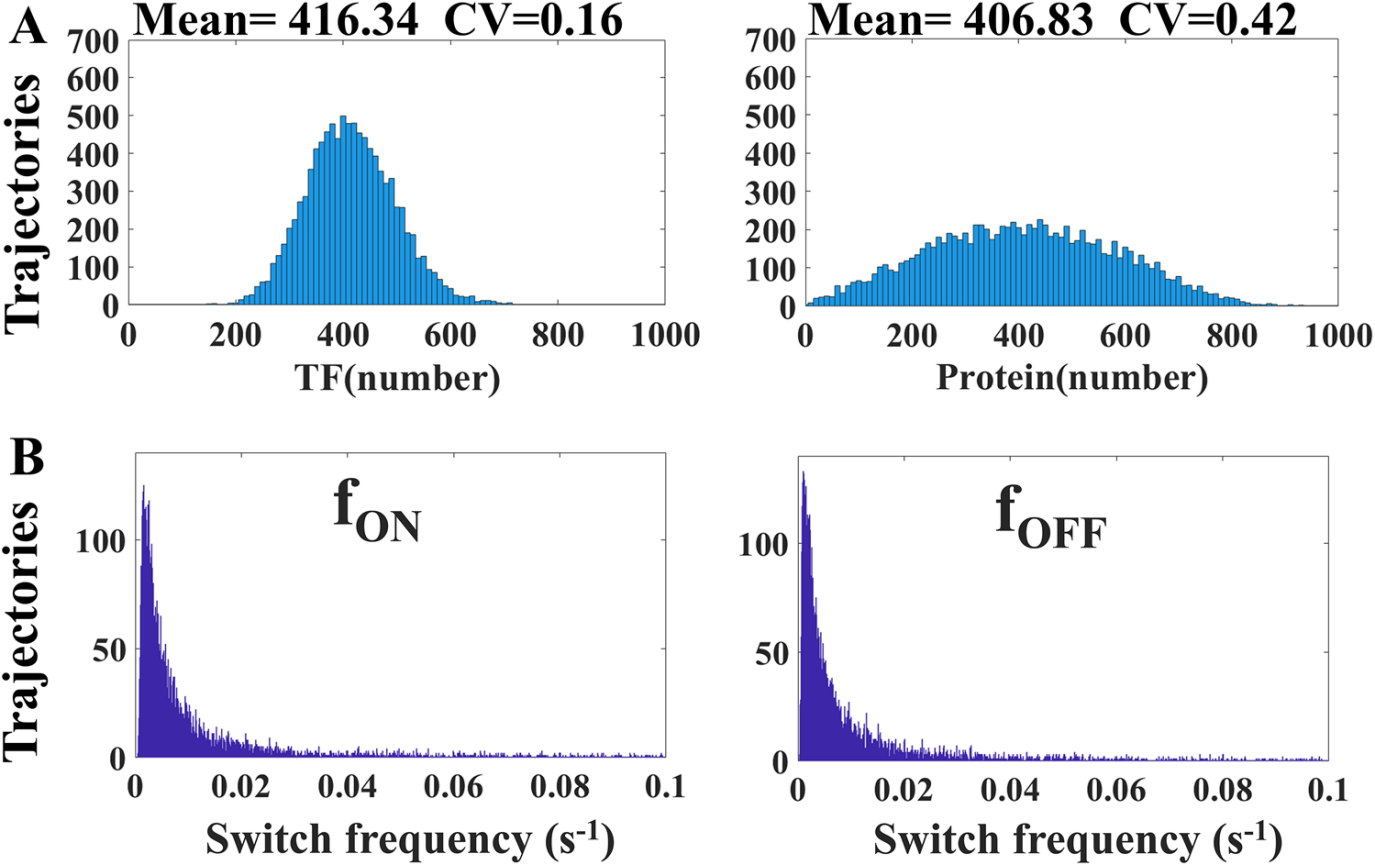
(**A**) shows the distribution of TF_B_ (left panel) and downstream protein (right panel). In comparison to the case in Fig 3A, lower value of TF_B_ leads to higher CV of the downstream protein. (**B**) shows the distribution of switching frequency of DNA.

This unique feature of DNA also results in another interesting phenomenon. In addition to Fig. 3 which illustrates how downstream protein responding to different noises of TF_B_, we then examined how different values of TF_B_ affect the protein distribution. Figure 5A shows the distribution of TF_B_ (left panel) and the protein (right panel). The CV of TF_B_ in Fig. 5A is nearly the same as that in Fig. 3A, but the distribution of the protein in Fig. 5A appeared to be much broader. Figure 5B shows the DNA switching frequency corresponding to the cases of Fig. 5A. In comparison to Fig. 4A, we realized that low switching frequency leads to high noise at the protein level. TF_B_ controls the switching frequency and the low value of TF_B_ leads to high protein noise.

### The influence of TF_E_ and TF_F_

**Figure 6:**
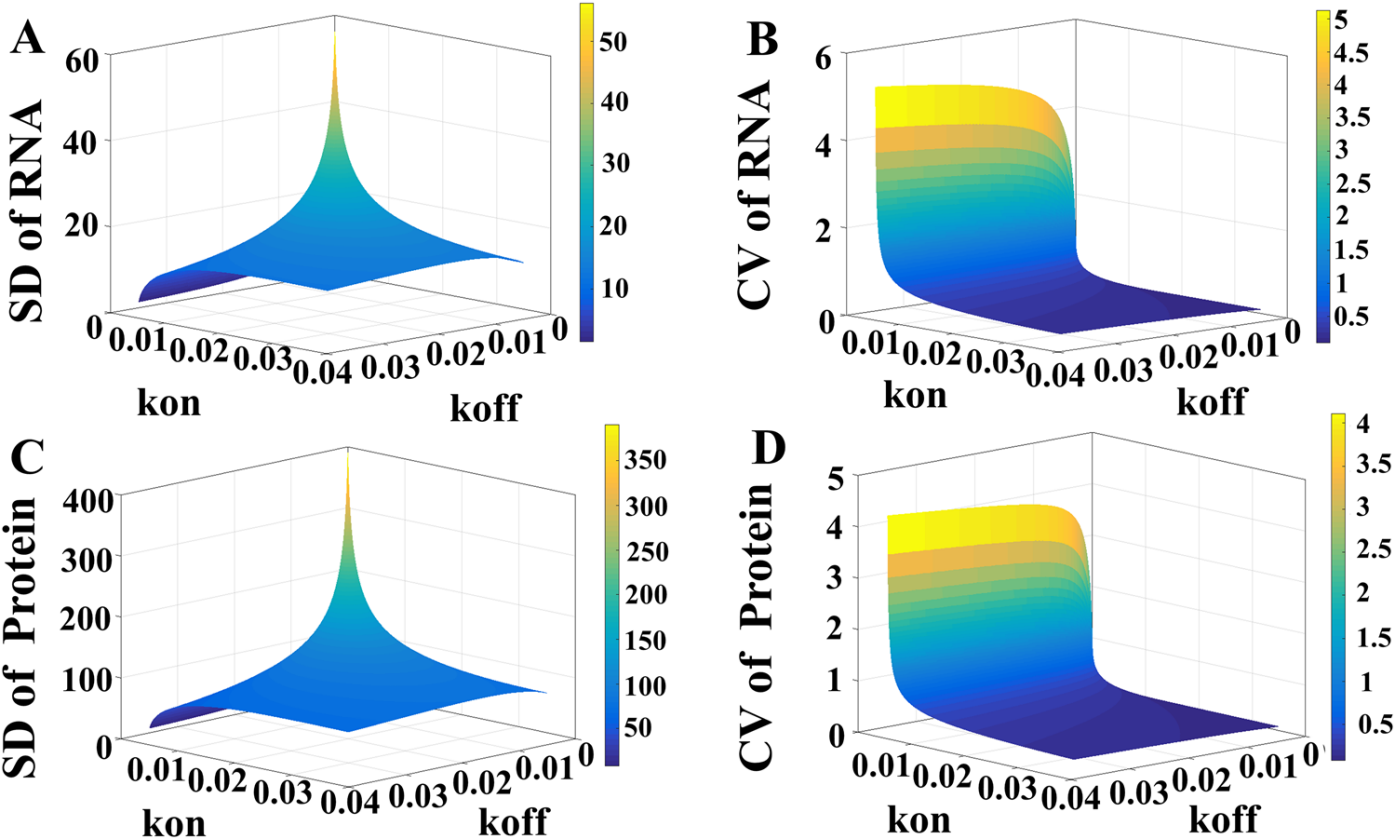
The SD and CV of RNA and protein with different values of k_on_ and k_off_. The rate constant k_off_ describes the switch of DNA from the ON to the OFF state and k_on_ is from the OFF to the ON states. (**A**,**B**) are the SD and CV of RNA, respectively. The SD and CV of protein are (**C**,**D**), respectively.

The transcription factors TF_E_ and TF_F_ control the rate constants of k_off_ and k_on_, respectively. By using the probability generating function (PGF), we explored the influence of k_off_ and k_on_ on the downstream elements. Figure 6 showed the standard deviation, SD, and the CV. As expected, the SD (Fig. 6A) and the CV (Fig. 6B) of RNA is large when both k_off_ and k_on_ are low. The behaviors of the protein (Fig. 6C,D) are similar to that of RNA. The results suggest that the DNA is capable of blocking out the upstream noise at high k_off_ and k_on_. High TF_B_ leads to low protein noise as the TF_B_ changes the system along with the line of k_off_ = k_on_. Also note that high k_on_ results in high mean value and low CV of protein. On the other hand, high k_off_ does not always grant a low CV of the protein.

## Discussion

In the present study, we illustrated that the broad distribution of TF in the FM system does not always lead to a huge noise of downstream protein (Fig. 3). This is because of the unique role of DNA which transforms the input of TF (the physical-domain representation) into switching frequency (the frequency domain representation) (Fig. 4). Some researchers are interested in a bimodal distribution of protein caused by a very low switching frequency of DNA^33^. Nevertheless, we worked on the TF concentration causing the switching frequency higher than the region of clear bimodality; low TF concentration leads to a low switching frequency and a broad distribution at protein level (Fig. 5). With Fig. 6, we know that the ability of DNA to block the noise is great at high k_on_ and k_off_.

Researchers who work on the binding between the transcription factor and DNA pointed out the k_on_ and k_off_ in *E. coli* are about 0.1–10 and 0.01–1 (1/s), respectively^34,35^. In comparison to Fig. 6, these values grant DNA the ability to block out the upstream noise. On the other hand, eukaryotes have lower rate constants^36,37^. As the volume of eukaryotes are much larger than that of prokaryotes, the intracellular components in eukaryotes usually suffer less noise and they may not rely on this mechanism. Besides, many processes in eukaryotes make difficult the observation of DNA resetting noise. They are processes of chromatin remodeling^38^, enhancers assisting TFs in accessing binding sites^39^, the exportation of mRNA to cytoplasm^7^, and so on^40^. The model, which incorporates many promoter states of chromatin remodeling and applies the same transcription rates to all ON states^41^, belongs to FM systems. While applying the concept of the present study, one should check the switching frequency of DNA in each promoter state. However, the system, which has different transcription rates for different promoter states, no longer belongs to FM systems but is more like an AM system.

It is important to aware that only DNA in the FM system is capable of resetting the noise. DNA in the AM system plainly passes the upstream noise to the downstream protein. This is because the DNA in the AM system has a ramp response rather than two states^2^. The expression level of DNA is capable of passing information regarding the distribution of upstream TF. In these systems, other regulatory strategies maybe employed to reduce the noise from TF and the most famous motif is the incoherent FFL. In addition, it is also possible to observe the application of the incoherent FFL to FM system with low k_on_ and k_off_^27^.

## Models

### The reaction networks

The reactions corresponding to networks in Fig. 1A,B are in Table S1 and S2, respectively. Table S3 is the nomenclature and Table S4 lists the nominal values of parameters. These values were used to generate Figs. 2A, 3A, and 4A. The formation of the TF was through the processes of transcription and translation. By changing the translation rate constant of TF, we tuned the noise intensity of the TF to generate Fig. 3B and 4B. Specifically, we increased the translation rate constant to four folds of the nominal value and decreased the transcription rate constant accordingly, to have the same protein level.

### The stochastic simulation

We conducted the numerical analysis by Stochastic Simulation Algorithm (SSA)^42^ with random number generator **rand** in Matlab. The distribution was composed of at least ten thousand trajectories. For each trajectory, we sampled the data at fifty thousand seconds. The system should already reach the steady state, as the mean and the variance of the distribution were time-independent. The initial condition for each intracellular variable is zero except for DNA that is one per cell. We used the typical size of E coli, 10^–15^ L, as cellular volume. We applied the binomial distribution to intracellular species^43^ to mimic the cell partitioning; the average time span is 30 min and is described by Gaussian distribution with the ratio of standard deviation to mean as 10%^44^.

### The probability generating function

We applied probability generating function (PGF) to the FM system. Note that we did not account for the distribution of transcription factors in PGF, but directly worked on k_on_ and k_off_. The details are in Text S1.

## Supplementary Information

Supplementary Information.
