# Supplementary material for: The switch of DNA states filtering the extrinsic noise in the system of frequency modulation
Source: Sci Rep. 2021 Aug 11;11:16309. doi: 10.1038/s41598-021-95365-0 (PMC8357933; doi:10.1038/s41598-021-95365-0)
Supplement: Supplementary file 1 — Supplementary Information. [file 41598_2021_95365_MOESM1_ESM.docx]

**The Switch of DNA States Filtering the Extrinsic Noise in the System of Frequency Modulation**

Shih-Chiang Lo, Chao-Xuan You, Bo-Ren Chen, Ching-Chu Hsieh, Cheng-En Li, Che-Chi Shu*

Department of Chemical Engineering and Biotechnology,
National Taipei University of Technology, Taiwan

**Supplementary Materials**

(The followings are based on the order mentioned in the main text.)

**Table S1: The reactions in the system of amplitude modulation (Fig 1A)**

|  |
| --- |
|  |
|  |
|  |
|  |
|  |
|  |
|  |

**Table S2: The reactions in the system of frequency modulation (Fig 1B)**

|  |
| --- |
|  |
|  |
|  |
|  |
|  |
|  |
|  |
|  |
|  |
|  |
|  |
|  |
|  |
|  |
|  |
|  |
|  |

**Table S3: Nomenclature of the variables**

| Annotation | Description |
| --- | --- |
| *DNAoff* | DNA of frequency modulation in inactive conformation |
| *DNAon* | DNA of frequency modulation in active conformation |
| *DNA* | DNA of amplitude modulation |
| *RNA* | RNA that produces protein, P |
| *P* | the very Protein in which we are interested |
| *DNATFA* | DNA of TF*A* |
| *DNATFB* | DNA of TF*B* |
| *DNATFF* | DNA of TF*F* |
| *DNATFE* | DNA of TF*E* |
| *RNATFA* | RNA from DNA*TFA* |
| *RNATFB* | RNA from DNA*TFB* |
| *RNATFF* | RNA from DNA*TFF* |
| *RNATFE* | RNA from DNA*TFE* |
| *TFA* | Transcription factor in system of amplitude modulation |
| *TFB* | Transcription factor controlling kon and koff |
| *TFF* | Transcription factor controlling kon |
| *TFE* | Transcription factor controlling koff |
|  | This symbol indicates the degradation |

**Table S4: The values of parameters**

| **Parameter** | **Description** | **Value** | **Units** | **Ref.** |
| --- | --- | --- | --- | --- |
| *kRTFA*a | Transcription rate constant of DNATFA | **9x10­­­­­-2** | S-1 | 1 |
| *kRTFBb* | Transcription rate constant of DNATFB | **6x10­­­­­-2** | S-1 | 1 |
| *kRTFF* | Transcription rate constant of DNATFF | **6x10­­­­­-2** | S-1 | 1 |
| *kRTFE* | Transcription rate constant of DNATFE | **6x10­­­­­-2** | S-1 | 1 |
| *kTFA*a | Translation rate constant of TFA | **5.6x10­­­­­-1** | S-1 | 1 |
| *kTFBb,c* | Translation rate constant of TFB | **5x10­­­­­-1** | S-1 | 1 |
| *kTFF* | Translation rate constant of TFF | **5x10­­­­­-1** | S-1 | 1 |
| *kTFE* | Translation rate constant of TFE | **5x10­­­­­-1** | S-1 | 1 |
| *krA* | Transcription rate constant of DNA | **1x10­­­­­-3** | Number-1S-1 | 1 |
| *kpA* | Translation rate constant of Protein P | **1x10­­­­­-3** | S-1 | 1 |
| *k1* | The rate of inversion from DNAoff to DNAon | **8.33 x10­­­­­-6** | Number-1S-1 | 2 |
| *k-1* | The rate of inversion from DNAon to DNAoff | **8.33 x10­­­­­-6** | Number-1S-1 | 2 |
| *kr* | Transcription rate constant of DNAon | **2.32x10­­­­­-1** | S-1 | 1 |
| *kp* | Translation rate constant of Protein P | **2x10­­­­­-2** | S-1 | 1 |
| *r* | Degradation rate constant of RNA | **2x10­­­­­-3** | S-1 | 3 |
| *g* | Degradation rate constant of Protein P | **2x10­­­­­-3** | S-1 | 3 |
| *kdRTFA* | Degradation rate constant of RNATFA | **6x10­­­­­-3** | S-1 | 3 |
| *kdRTFB* | Degradation rate constant of RNATFB | **6x10­­­­­-3** | S-1 | 3 |
| *kdRTFF* | Degradation rate constant of RNATFF | **6x10­­­­­-3** | S-1 | 3 |
| *kdRTFE* | Degradation rate constant of RNATFE | **6x10­­­­­-3** | S-1 | 3 |
| *kdTFA* | Degradation rate constant of Protein TFA | **2x10­­­­­-3** | S-1 | 3 |
| *kdTFB* | Degradation rate constant of Protein TFB | **2x10­­­­­-3** | S-1 | 3 |
| *kdTFF* | Degradation rate constant of Protein TFF | **2x10­­­­­-3** | S-1 | 3 |
| *kdTFE* | Degradation rate constant of Protein TFE | **2x10­­­­­-3** | S-1 | 3 |

[1] Shu, C. C., Yeh, C. C., Jhang, W. S., & Lo, S. C. (2016). Driving cells to the desired state in a bimodal distribution through manipulation of internal noise with biologically practicable approaches. PloS one, 11(12), e0167563.

[2] Hung, M., Chang, E., Hussein, R., Frazier, K., Shin, J. E., Sagawa, S., & Lim, H. N. (2014). Modulating the frequency and bias of stochastic switching to control phenotypic variation. Nature communications, 5(1), 1-11.

[3] Osella, M., Bosia, C., Corá, D., & Caselle, M. (2011). The role of incoherent microRNA- mediated feedforward loops in noise buffering. PLoS computational biology, 7(3), e1001101.

a. Fig.2A, we increased kRTFA by two folds and decreased kTFA accordingly to keep the protein level and reduce the TF noise. On the other hand, in Fig.2B, we increased kTFA by three folds and decreased kRTFA accordingly to keep the protein level.

b. Fig.3B, we increased *kTFB* by 4 folds and decreased *kRTFB* accordingly to keep the protein level.

c. For low switching frequency in Fig. 5 we decreased *kRTFB* to 0.01.

**Text S1: The Probability Generating Function**

We accounted for the system of gene network with following reactions.

For the sake of simplicity, we use variables n1, n2, n3 and n4 instead of DNAoff, DNAon, RNA and P. We use $p(\mathbf{n}\mathbf{,}t)$ to describe the probability of the system with states **n**=(n1, n2, n3, n4) at time t. Note that the number of total DNA is unity, namely $DNA_{on}+DNA_{off}=1$, so we use rather than $p\left( \mathbf{n}\mathbf{,}t \right)$. We have the Chemical master equation as the following.

We then introduce the generating function,

$F(z_{2},z_{3},z_{4},t)=\sum_{n_{2},n_{3},n_{4}=0}^{\infty} z_{2}^{n_{2}}z_{3}^{n_{3}}z_{4}^{n_{4}}p(n_{2},n_{3},n_{4},t)$.

There are some general properties

$$\left.F\right|_{1}\equiv\text{ }1,\text{ }\left.\frac{\partial F}{\partial z_{i}}\right|_{1}\equiv\text{ }<n_{i}>,\text{ }\left.\frac{\partial^{2}F}{\partial z_{i}^{2}}\right|_{1}\equiv\text{ }<n_{i}(n_{i}-1)>=<n_{i}^{2}>-<n_{i}>,\text{ }\left.\frac{\partial^{2}F}{\partial z_{i}\partial z_{j}}\right|_{1}\equiv\text{ }<n_{i}n_{j}>$$

, where the angled bracket denotes the mean and notation |1 means that the function is evaluated at zj = 1 for all j. We obtained the mean of the RNA and protein, which are n3 and n4 respectively.

$$<RNA>=\frac{k_{r}}{r}<DNA_{on}>$$

$$<P>=\frac{k_{p}}{g}<RNA>$$

To have the variance of RNA, we calculated $\left.\frac{\partial^{2}F}{\partial z_{3}^{2}}\right|_{1}\equiv\text{ }<n_{3}^{2}>-<n_{3}>$, and it needs $<n_{2}n_{3}>$ which can be obtained by $\left.\frac{\partial^{2}F}{\partial z_{2}\partial z_{3}}\right|_{1}$ . To calculate $\left.\frac{\partial^{2}F}{\partial z_{2}\partial z_{3}}\right|_{1}$ , it needs $<n_{2}^{2}>$which can be obtained by $\left.\frac{\partial^{2}F}{\partial z_{2}^{2}}\right|_{1}+\text{ }\left.\frac{\partial F}{\partial z_{2}}\right|_{1}$. Finally, we have the standard deviations of RNA,

$\sigma_{RNA}=\sqrt{\frac{k_{r}}{r}\cdot\frac{k_{on}<RNA>+k_{r}<DNA_{on}^{2}>}{k_{off}+k_{on}+r}+\frac{k_{r}}{r}<DNA_{on}>-(\frac{k_{r}}{r})^{2}<DNA_{on}>^{2}}$.

In order to have the variance of protein which is $\left.\frac{\partial^{2}F}{\partial z_{4}^{2}}\right|_{1}\equiv\text{ }<n_{4}^{2}>-<n_{4}>$, we need the $\left.\frac{\partial^{2}F}{\partial z_{3}\partial z_{4}}\right|_{1}\equiv\text{ }<n_{3}n_{4}>$, $\left.\frac{\partial^{2}F}{\partial z_{2}\partial z_{4}}\right|_{1}\equiv\text{ }<n_{2}n_{4}>$, and $\left.\frac{\partial^{2}F}{\partial z_{2}\partial z_{3}}\right|_{1}\equiv\text{ }<n_{2}n_{3}>$. We then have the standard deviations of protein,

$$\sigma_{P}=\sqrt{\left\{\left.\left(\frac{k_{p}}{g}\right)\left[\left(\frac{k_{r}}{g+r}\right)\left(\frac{k_{on}}{k_{on}+k_{off}+g}<P>+\frac{k_{p}}{k_{on}+k_{off}+g}\left(\frac{k_{r}\text{<}DNA_{on}^{2}\text{> + }k_{on}<RNA>}{k_{on}+k_{off}+r}\right)\right)+\left(\frac{k_{p}}{g+r}\right)<RNA^{2}>\right]+<P>\right\}\right.-<P>^{2}}$$
